# Frailty’s influence on 30-day mortality in old critically ill ICU patients: a bayesian analysis evaluating the clinical frailty scale

**DOI:** 10.1186/s13613-023-01223-9

**Published:** 2023-12-13

**Authors:** Bernhard Wernly, Raphael Romano Bruno, Michael Beil, Hans Flaatten, Malte Kelm, Sviri Sigal, Wojciech Szczeklik, Muhammed Elhadi, Michael Joannidis, Andreas Koköfer, Sandra Oeyen, Brian Marsh, Rui Moreno, Sarah Wernly, Susannah Leaver, Dylan W. De Lange, Bertrand Guidet, Christian Jung

**Affiliations:** 1 Institute of General Practice, Family Medicine and Preventive Medicine, Paracelsus Medical University of Salzburg, 5020 Salzburg, Austria; 2 Department of Internal Medicine, General Hospital Oberndorf, Teaching Hospital of the Paracelsus Medical University Salzburg, 5020 Salzburg, Austria; 3 Medical Faculty, Department of Cardiology, Pulmonology and Vascular Medicine, Heinrich-Heine-University Duesseldorf, 40225 Düsseldorf, Germany; 4 Department of Medical Intensive Care, Hadassah Medical Center and Faculty of Medicine, Hebrew University of Jerusalem, 91120 Jersualem, Israel; 5 Department of Clinical Medicine, University of Bergen, Department of Anaestesia and Intensive Care, Haukeland University Hospital, 5021 Bergen, Norway; 6 Cardiovascular Research Institute Düsseldorf (CARID), Medical Faculty, Heinrich-Heine University, Duesseldorf, Germany; 7 Center for Intensive Care and Perioperative Medicine, Jagiellonian University Medical College, 31-008 Krakow, Poland; 8 Faculty of Medicine, University of Tripoli, R6XF+46G, Tripoli, Libya; 9 Division of Intensive Care and Emergency Medicine, Department of Internal Medicine, Medical University Innsbruck, 6020 Innsbruck, Austria; 10 Department of Anaesthesiology, Perioperative Medicine and Intensive Care, Paracelsus Medical University of Salzburg, 5020 Salzburg, Austria; 11 Department of Intensive Care 1K12IC, Ghent University Hospital, 9000 Ghent, Belgium; 12 Mater Misericordiae University Hospital, Dublin, D07 R2WY Ireland; 13 Centro Hospitalar de Lisboa Central, Faculdade de Ciências Médicas de Lisboa, Nova Medical School, Lisboa, Portugal; 14 Faculdade de Ciências da Saúde, Universidade da Beira Interior, Covilhã, Portugal; 15 General Intensive Care, St. George´S University Hospital NHS Foundation Trust, London, SW17 0QT UK; 16 Department of Intensive Care Medicine, University Medical Center, University Utrecht, 3584 CX Utrecht, Utrecht, The Netherlands; 17 Inserm, Service de‘Réanimation, Sorbonne Université, Hôpital Saint-Antoine, Institut Pierre-Louis d‘épidémiologie Et de Santé Publique, AP-HP, 184, Rue du Faubourg-Saint-Antoine, 75012 Paris, France; 18 Division of Cardiology, Pulmonology and Vascular Medicine, University Duesseldorf, Moorenstraße 5, 40225 Duesseldorf, Germany

## Abstract

**Introduction:**

Frailty is widely acknowledged as influencing health outcomes among critically ill old patients. Yet, the traditional understanding of its impact has predominantly been through frequentist statistics. We endeavored to explore this association using Bayesian statistics aiming to provide a more nuanced understanding of this multifaceted relationship.

**Methods:**

Our analysis incorporated a cohort of 10,363 older (median age 82 years) patients from three international prospective studies, with 30-day all-cause mortality as the primary outcome. We defined frailty as Clinical Frailty Scale ≥ 5. A hierarchical Bayesian logistic regression model was employed, adjusting for covariables, using a range of priors. An international steering committee of registry members reached a consensus on a minimal clinically important difference (MCID).

**Results:**

In our study, the 30-day mortality was 43%, with rates of 38% in non-frail and 51% in frail groups. Post-adjustment, the median odds ratio (OR) for frailty was 1.60 (95% CI 1.45–1.76). Frailty was invariably linked to adverse outcomes (OR > 1) with 100% probability and had a 90% chance of exceeding the minimal clinically important difference (MCID) (OR > 1.5). For the Clinical Frailty Scale (CFS) as a continuous variable, the median OR was 1.19 (1.16–1.22), with over 99% probability of the effect being more significant than 1.5 times the MCID. Frailty remained outside the region of practical equivalence (ROPE) in all analyses, underscoring its clinical importance regardless of how it is measured.

**Conclusions:**

This research demonstrates the significant impact of frailty on short-term mortality in critically ill elderly patients, particularly when the Clinical Frailty Scale (CFS) is used as a continuous measure. This approach, which views frailty as a spectrum, enables more effective, personalized care for this vulnerable group. Significantly, frailty was consistently outside the region of practical equivalence (ROPE) in our analysis, highlighting its clinical importance.

## Introduction

The global demographic shift towards an increasingly older population has significantly increased the number of critically ill older patients requiring intensive care. According to estimates from the World Health Organization (WHO), the population aged 60 years and over is expected to nearly double by 2050, a significant portion of whom will inevitably require intensive care due to age-related medical conditions [1]. This presents a unique set of challenges for healthcare providers, as the intensive care needs and management of these older patients differ from their younger counterparts. Specifically, the high prevalence of comorbidities, polypharmacy, and overall physiological decline are all factors that need to be considered in the critical care of older patients [2].

Frailty, a state of increased vulnerability to stressors due to age-associated declines in physiologic reserves, is emerging as a key concept in evaluating and managing older, critically ill patients [2–4]. Previous studies have underscored the importance of frailty in predicting adverse outcomes, such as increased hospitalization, functional decline, and mortality in this population [5, 6]. The Clinical Frailty Scale (CFS) is a tool commonly used to measure frailty, providing a simple and reliable estimate of the vulnerability of older individuals [3, 7]. Its integration into routine clinical assessments has been associated with more accurate prognostication and might lead to better-informed clinical decision-making [8]. The assessment of frailty plays a critical role in the intensive care of older patients for several reasons. Primarily, it serves as an important tool for risk stratification, enabling healthcare providers to identify those who are most vulnerable and likely to have poor outcomes. This ability to stratify risk facilitates appropriate resource allocation and tailoring of treatment strategies, helping to ensure that the level of care provided aligns with patient needs and prognosis. In addition, frailty provides a critical context for defining realistic therapeutic goals, potentially beyond information about the acute illness [9]. Conversations about treatment expectations and prognosis are pivotal in the intensive care setting [10]. By understanding a patient's level of frailty, physicians can engage in more informed discussions with patients and their families about what to expect and what goals are realistic. This promotes shared decision-making and allows for alignment of care with the patient's values and preferences. Given these significant implications, a thorough understanding of the effects of frailty on mortality and the magnitude of these effects is crucial. It allows for better prognostication, more appropriate goal-setting, and ultimately, more personalized and effective care for critically ill old patients.

However, despite the recognized importance of frailty assessment and the growing body of literature supporting its prognostic value, existing studies primarily employ frequentist statistical approaches [5, 6, 11, 12]. Here, logistic regression is particularly suitable for binary outcomes, and one of its most distinctive features is that it produces odds ratios as measures of effect. However, odds ratios are not intuitively easy to understand [13]. An odds ratio is a measure of association between an exposure and an outcome, indicating the odds that an outcome will occur given a particular exposure, compared to the odds of the outcome occurring in the absence of that exposure.

Furthermore, while frequentist statistical approaches have provided valuable insights, they have limitations, particularly when dealing with uncertainties inherent in clinical research. Unlike Bayesian statistics, frequentist methods estimate the likelihood of observed data given a specific hypothesis but do not provide a probability for the hypothesis itself [14]. Bayesian methods, on the other hand, allow for the estimation of the probability of a hypothesis given observed data, offering an intuitive and straightforward way to understand the likely magnitude of an effect. This can be particularly useful in the context of frailty on the ICU, where patient-specific factors influence the outcome of applied therapies [14]. With this Bayesian approach, we can provide clinicians with more nuanced, patient-specific, and intuitive probabilities regarding the impact of frailty on outcomes, such as 30-day mortality.

This study aimed to analyze the association between frailty and 30-day mortality using measures of association that are intuitively understandable. Specifically, we employed a Bayesian methodology. In summary, we aim to present a comprehensive and, most importantly, an intuitive understanding of the association between frailty and 30-day mortality.

## Methods

### Patients

We included patients of three registries (VIP-1, VIP-2 and COVIP) in this analysis [5, 6, 11]. In short, these trials prospectively enrolled old critically ill patients during three timeframes. We included 3830 patients from VIP-1 (timeframe between October 2016 to May 2017), 377o from VIP-2 (timeframe between May 2018 and May 2019) and 2754 from COVIP-1 (timeframe between March 2020 to May 2021) We included all unique acute admissions (*n* = 10,363) with complete data on 30-day mortality, CFS, age, sex, SOFA score at admission, treatment limitation decisions, and country of inclusion. Missing data were < 1%. We specifically excluded 935 patients from VIP-1 who were admitted to the ICU after elective surgery.

### Statistical analysis

All statistical analyses were performed using Stata/BE 18.

### Minimal clinically important difference

We surveyed the 24 members of the international steering committee of the VIP network to generate consensus on a MCID for the primary endpoint and the primary (frailty as binary variable; CFS ≥ 5) and the secondary (frailty as continuous variable per CFS point) exposure. To obtain estimates for MCID, two questions were emailed to the international Steering Committee members by Christian Jung. Members were asked to rank their three top choices. Of the 24 steering committee members, 17 returned their answers. We defined a priori that the MCID would be defined using the following decision rules: (1) if the most frequent option elected as the first option was also the most frequent option that received any vote, that option would represent MCID by consensus. (2) If the most frequently voted first choice was not the option that received more overall votes, we would define that no clear consensus occurred and would display both options.

An absolute difference of 10% over the baseline event rate of 40% for the primary endpoint, 30-day mortality, was considered as the MCID (which results in an odds ratio of approximately 1.5). Therefore, the margin for a large effect was set as 1.5 × above 1.84 as previously suggested [15].

### Region of practical equivalence (ROPE)

We defined the region of practical equivalence for both the primary (frailty, defined as CFS ≥ 5) and secondary (CFS as a continuous variable) exposure. For the primary exposure, we established the ROPE using thresholds that identified an effect as “significant.” as previously suggested by Cohen and Kruschke [15–18]. These thresholds corresponded to a difference in the logarithm of the odds ratio (log(OR)) equivalent to a standardized mean difference of 0.1 on Cohen’s d scale. This specific threshold equates to a log(OR) difference of 0.18. To convert from Cohen’s d to a standardized log(OR), the log(OR) is multiplied by (Pi * the square root of three). Consequently, this translates to an odds ratio ranging from 0.83 to 1.19. For the secondary exposure, we defined the ROPE to be between OR 1.1 and 1/1.2 based on the considerations of Zampieri [14].

### Bayesian analysis

To evaluate the associations between the primary endpoint 30-day mortality and the primary exposure (frailty, defined as CFS ≥ 5) and the secondary exposure (CFS as a continuous variable), we fitted models using multilevel Bayesian logistic regression using the country of inclusion as a random intercept. Model-1 was the univariate association between the exposure and 30-day mortality. We further consider model-1 to be an oversimplification and only exploratory and consider model-2 as our primary model of interest. For model-2, we adjusted for age, sex, SOFA score at admission, and the admission diagnosis. For the model-3, we added the presence of a treatment limitation to the model-2. Model-3 was only exploratory as it included non-baseline information. We consider it very likely that this model is overcorrected as treatment limitations might be one of the mediators in the frailty and 30-day mortality association. We consider the aim of this study to be etiological (rather than a prediction system) [19].

We used three priors and developed them based on the principles proposed by Zampieri et al. [14]. First, we defined the priors for the primary exposure. We established a neutral or "skeptical" prior with parameters (log(1), 0.36), operating under the assumption that there is no effect from the exposures, denoted mathematically as OR = 1. The standard deviation (SD) was selected to encompass 95% of the probability between an odds ratio (OR) of 0.5 and 2, resulting in an SD of 0.36. In addition, we formulated an optimistic prior with parameters (log(0.66), 0.79), which implies an expectation that frailty is linked to better outcomes, represented by an OR of 0.66. Given existing evidence and our clinical judgment, we viewed this expectation as weak and thus set the SD to allow for a 30% chance of harm (OR > 1), yielding an SD of 0.79. A pessimistic prior was also set with parameters (log(1.5), 0.25), expressing a strong belief that frailty leads to worse outcomes, symbolized by an OR of 1.5. The SD for this belief was calculated to permit a 5% chance of benefit (OR < 1), leading to an SD of 0.25.

The Clinical Frailty Scale (CFS) is treated as a continuous variable. These included a flat neutral prior (log(1), 1), a weak optimistic prior (log(0.9), 0.20), and a strong pessimistic prior (log(1.1), 0.06). We then computed the posterior odds ratios along with their 95% credibility intervals (CrI), as well as the probabilities of harm (OR > 1) and significant harm (OR exceeding the minimal clinically important difference, or MCID, which is OR > 1.5 for CFS ≥ 5 and OR > 1.04 per CFS point), and also for a large effect (1.5 times MCID). All statistical procedures were carried out using Stata/BE 18.

### Frequentist analysis

Continuous data were analyzed using the frequentist Mann–Whitney *U* test or student's *T* test, depending on the distribution of the data, and are presented as median ± interquartile range (IQR) or mean ± standard deviation (SD), respectively. Categorical data are presented as numbers (percentage) and were compared using the Chi-square test. All tests were two-tailed, and a *p* value of < 0.05 was considered statistically significant.

## Results

The baseline characteristics of the study population are shown in Table 1. The median SOFA score was 6 points. The median age was 82 years. The overall 30-day mortality was 43%, the mortality in the non-frail group 38%, and 51% in the frail group.

**Table 1:** Posterior distribution of frailty (CFS ≥ 5) being associated with 30-day mortality after multivariable adjustment and using the skeptical prior.

	CFS < 5	CFS ≥ 5	*p* value
	*N* = 6481	*N* = 3882	
Age (years)	81 (6)	84 (5)	< 0.001
SOFA (points)	6 (4)	7 (4)	< 0.001
Female	38% (2448)	51% (1993)	< 0.001
NIV (yes/no)	24% (1523)	27% (1060)	< 0.001
Mechnical ventilation (yes/no)	59% (3809)	51% (1987)	< 0.001
Vasoactive drugs (yes/no)	61% (3972)	60% (2322)	0.14
Treatment limitations (yes/no)	30% (1970)	41% (1594)	< 0.001
30-day survival (yes/no)	38% (2446)	51% (1964)	< 0.001
Amount of comorbidites (n) *	4 (2)	5 (3)	< 0.001
* available in 3913 patients			

The median OR was 1.60 and the corresponding 95% credible interval 1.45–1.76

**Fig. 1:**
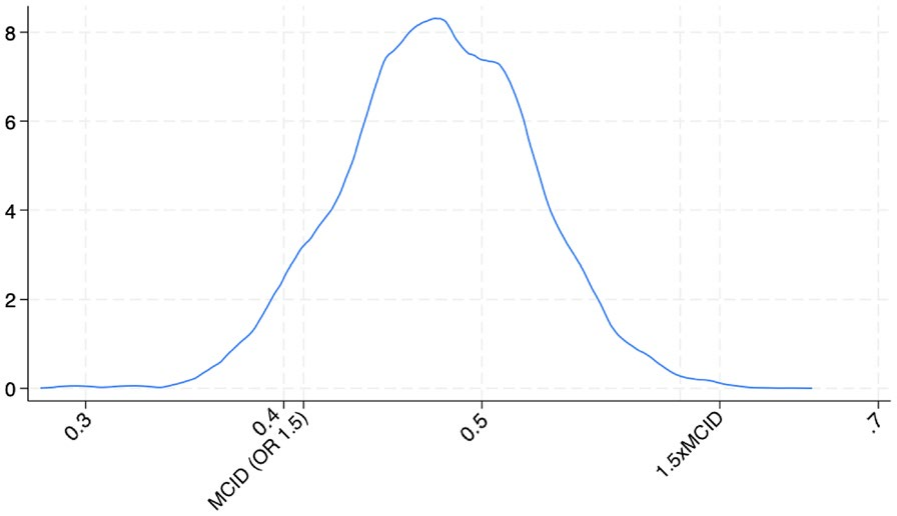
Posterior distribution of the log odds ratio (OR) using an optimistic prior (− 0.42,0.79) which represents the belief that frailty is associated with better outcome. The distribution represents 10,000 draws from the posterior, which approximates to a normal distribution with a mean of 0.47 which corresponds to an OR of 1.60. The vertical line at 0.41 corresponds to the MCID (OR of 1.5) and the vertical line at 0.61 to 1.5 × MCID. This graphically represents the high likelihood of the OR being greater than the MCID, and the low likelihood of the OR being smaller than 1.5 × MCID

The posterior probability distribution of the odds ratios of the primary exposure frailty (CFS ≥ 5) with the primary endpoint of 30-day mortality is shown in Table 2. After multivariable adjustment and using the “optimistic” prior (representing the belief that frailty is associated with better outcomes), the median OR was 1.60, and the corresponding 95% credible interval 1.45–1.76 (Fig. 1). Even with the optimistic prior the probability for frailty being associated with harm (OR > 1) was > 99%, and the probability for the effect being OR > 1.5 was 90% (Table 2). This association of frailty with harm was consistent across all priors and after multivariable adjustment (Table 2). Similarly, the probability of the effect being larger than the established MCID (OR > 1.5) was above 90% regardless of the prior used in the primary model-2. After additional adjustment for the presence of treatment limitations (model-3), the probability of the effect being larger than the MCID was < 1%. The probability that the effect of frailty was > 1.5xMCID was < 1% under all assumptions. Similarly, under every considered assumption, the probability of the true effect falling within the ROPE was consistently 0%. In sensitivity analysis using skeptical priors, in male (median OR 1.76; 95% CI 1.58–1.98; probability of > MCID 0.99; probability of being within ROPE 0%) and female (median OR 1.59; 95%CI 1.40–1.80; probability > MCID 78% and being within ROPE 0%) as well as in patients without treatment limitations (median OR 1.71; 95% CI 1.52–1.92; probability > MCID 98% and being within ROPE 0%) yielded similar results. Of note, adjusting model-1 with the amount of comorbidities, did not alter the association of frailty with 30-day mortality (skeptical prior: median OR 1.48; 95%CI 1.28–1.69; probability being associated with harm > 99% and > MCID 39% and being within ROPE 0%).

**Table 2:** Posterior probability distribution of the odds ratios for the primary exposure, frailty (CFS ≥ 5), in relation to the primary endpoint, 30-day mortality

		OR (95% CrI)	Probability of Harm (OR > 1)	Probability of MCID (OR > 1.5)	Probability of large effect (1.5xMCID)
	Prior				
Model-1	Skeptical	1.62 (1.48–1.76)	> 0.99	0.94	< 0.01
	Optimistic	1.62 (1.49–1.76)	> 0.99	0.95	< 0.01
	Pessimistic	1.62 (1.49–1.77)	> 0.99	0.95	< 0.01
Model-2	Skeptical	1.60 (1.45–1.76)	> 0.99	0.89	< 0.01
	Optimistic	1.60 (1.46–1.75)	> 0.99	0.90	< 0.01
	Pessimistic	1.61 (1.47–1.77)	> 0.99	0.92	< 0.01
Model-3	Skeptical	1.29 (1.16–1.44)	> 0.99	< 0.01	< 0.01
	Optimistic	1.29 (1.16–1.44)	> 0.99	< 0.01	< 0.01
	Pessimistic	1.29 (1.16–1.42)	> 0.99	< 0.01	< 0.01

The posterior probability distribution of the odds ratios of the secondary exposure frailty (per CFS point) with the primary endpoint 30-day mortality are shown in Table 3. After multivariable adjustment and using the skeptical prior, the median OR was 1.19, and the corresponding 95% credible interval was 1.16–1.22. Even with the optimistic prior, the probability of frailty being associated with harm (OR > 1) was > 99%, and the probability of the effect being OR > 1.5 was > 99% (Table 2). This association of frailty with harm was consistent across all priors and after multivariable adjustment (Table 3). Similarly, the probability of the effect being larger than the established MCID (OR > 1.5) was above 99% regardless of the prior used in the primary model-2. Even after additional adjustment for the presence of treatment limitations (model-3), the probability of the effect being larger than the MCID was > 99%. The probability that the effect of frailty was > 1.5xMCID was > 99% under all assumptions. Again, likewise, the probability of the true effect being within the ROPE was 0% under all assumptions. In a sensitivity analysis employing skeptical priors, similar results were observed for both males (median OR 1.19; 95% CI 1.15–1.24; likelihood of exceeding MCID 99%; probability of falling within ROPE 0%) and females (median OR 1.20; 95% CI 1.17–1.24; probability of exceeding MCID > 99%; being within ROPE at 0%). This pattern was also seen in patients without treatment limitations (median OR 1.19; 95% CI 1.15–1.23; probability of exceeding MCID > 99% and being within ROPE 0%). Again, adjusting model-1 with the amount of comorbidities, did not alter the association of frailty with 30-day mortality (skeptical prior: median OR 1.17; 95%CI 1.13–1.2; probability being associated with harm > 99% and > MCID 99% and being within ROPE 0%).

**Table 3:** Posterior probability distribution of the odds ratios for the secondary exposure, frailty (per CFS point), in relation to the primary endpoint, 30-day mortality

		OR (95% CrI)	Probability of Harm (OR > 1)	Probability of MCID (OR > 1.5)	Probability of large effect
	Prior				
Model-1	Skeptical	1.18 (1.16–1.21)	> 0.99	> 0.99	> 0.99
	Optimistic	1.18 (1.16–1.21)	> 0.99	> 0.99	> 0.99
	Pessimistic	1.18 (1.16–1.21)	> 0.99	> 0.99	> 0.99
Model-2	Skeptical	1.19 (1.16–1.22)	> 0.99	> 0.99	> 0.99
	Optimistic	1.19 (1.16–1.22)	> 0.99	> 0.99	> 0.99
	Pessimistic	1.19 (1.16–1.22)	> 0.99	> 0.99	> 0.99
Model-3	Skeptical	1.11 (1.08–1.14)	> 0.99	> 0.99	> 0.99
	Optimistic	1.12 (1.09–1.15)	> 0.99	> 0.99	> 0.99
	Pessimistic	1.19 (1.07–1.14)	> 0.99	> 0.99	> 0.99

## Discussion

**Fig. 2:**
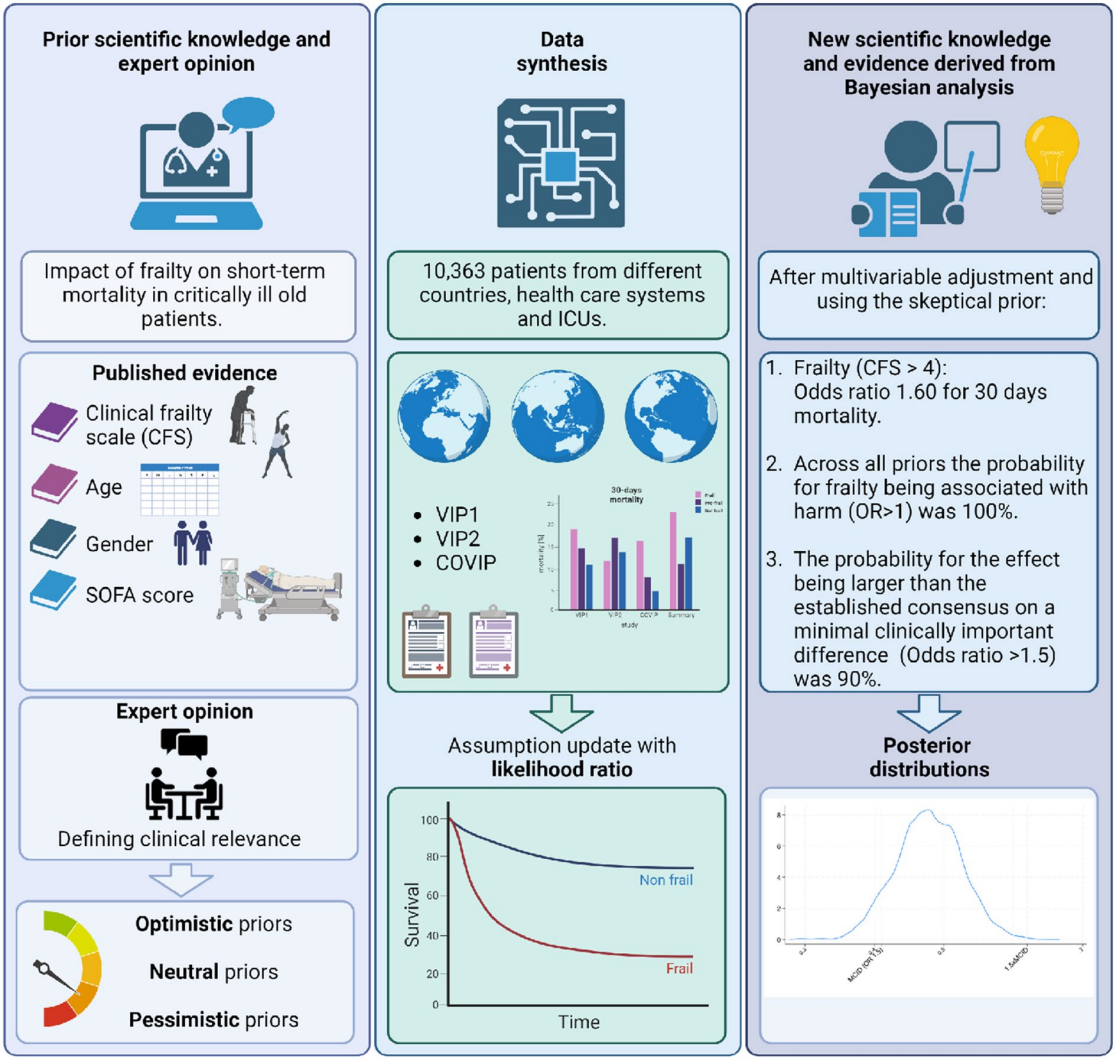
In our research, we assessed the relationship between frailty—quantified using the Clinical Frailty Scale (CFS)—and 30-day mortality in critically ill elderly patients. The observed association was pronounced, illuminating the pivotal role that frailty might have in forecasting short-term mortality within this demographic. To quantify the likelihood of this association and to estimate the effect size, we employed Bayesian modeling, providing a more nuanced understanding of the probabilities that frailty is linked with adverse outcomes. Recognizing this relationship is imperative; as it not only deepens our understanding of the associated risk factors but also empowers clinicians to deliver more tailored care

The present study assessed the relationship between frailty—quantified using the Clinical Frailty Scale (CFS)—and 30-day mortality in critically ill old patients (Fig. 2). The observed association was pronounced, illuminating the pivotal role of frailty in forecasting short-term mortality within this demographic. To quantify the likelihood of this association and estimate the effect size, we employed Bayesian modeling, providing a more nuanced understanding of the probability that frailty is linked with adverse outcomes. Recognizing this relationship is imperative, as it not only deepens our understanding of the associated risk factors and empowers clinicians to deliver more tailored care [2, 20].

Our study provides robust evidence indicating the influence of frailty, measured both by a binary CFS cutoff (CFS ≥ 5) and as a continuous variable (per CFS point), on the 30-day mortality in critically old ill patients. This relationship remains significant even after adjusting for numerous covariates, including sex, age, acute illness severity as well as comorbidities and in sensitivity analyses. Notably, the impact of frailty consistently surpasses the pre-established minimal clinically important difference (MCID), affirming its clinical relevance in predicting short-term mortality outcomes. Furthermore, under all conditions evaluated in our study, frailty was consistently outside the region of practical equivalence (ROPE). This observation further supports the clinical relevance of frailty, regardless of whether it is defined as a binary or a continuous variable. These findings reinforce the clinical utility of frailty, as measured by the CFS, in prognosticating and managing old, critically ill patients.

Traditional frequentist statistics, while providing an estimate of effect size and its statistical significance, do not offer straightforward information about the probability of the observed effect given the data. Bayesian methods, on the other hand, allow for direct computation of the probability that the effect size exceeds a given clinical threshold, such as the MCID. This probabilistic perspective yields more intuitive, patient-specific information, which is invaluable in complex, high-stakes clinical settings, such as critical care. In addition, a primary advantage of Bayesian methods is their ability to provide probabilities relating to the magnitude of effects. This enhances the depth of data analysis, offering a more nuanced understanding that can be particularly valuable in interpreting clinical research. Such detailed probabilistic insights are a significant contribution to the existing literature, particularly in areas, where understanding the scale and implications of effects is crucial.

While Bayesian analysis has traditionally been utilized in re-evaluating randomized controlled trials, its applicability extends significantly into the realm of observational studies, such as ours examining the relationship between CFS and 30-day mortality [14]. There are several reasons for this. First, observational studies often tackle real-world situations, where randomization may not be feasible or ethical, providing a unique perspective that complements insights gained from randomized trials. Bayesian methods are particularly suited for these settings as they can more flexibly accommodate the complexities of real-world data, including unbalanced designs, missing data, and non-normal distributions. They allow for the integration of prior information, either from previous studies or subject matter knowledge, which can be incredibly useful when dealing with complex, heterogeneous patient populations, such as the critically ill elderly. Moreover, Bayesian methods provide intuitive, probabilistic results that facilitate the understanding and communication of study findings. Given the observed data, they offer direct estimates of the probability of a hypothesis (in this case, the effect of CFS on 30-day mortality). This contrasts with frequentist statistics, which only provide the probability of the observed data under a specific hypothesis. In the context of our study, the Bayesian approach enables us to directly quantify the uncertainty about the size of the frailty effect and evaluate the likelihood that it exceeds clinically important thresholds. In the specific case of our study, Bayesian analysis can provide valuable insights into the effect of frailty on 30-day mortality after adjusting for confounders and treatment decisions. As we have found, these factors significantly influence the relationship between frailty and mortality, making Bayesian methods' ability to handle complex models especially beneficial. Thus, Bayesian analysis can play a pivotal role in unveiling the nuanced, real-world implications of frailty in critical care medicine.

In the context of our study's limitations, it is important to acknowledge the findings from one of our multivariable models—model-3—which, upon adjusting for treatment limitations, revealed that the probability of the frailty effect on 30-day mortality exceeding the minimal clinically important difference (MCID) was virtually nil. However, these results should be interpreted cautiously and considered thesis-generating rather than definitive conclusions. The rationale behind this cautious interpretation lies in our presumption that frailty's impact on short-term mortality may also be mediated via treatment limitations. Hence, a model that adjusts for treatment limitations might overcorrect the relationship between frailty and mortality. While the model appears to diminish the effect of frailty, it is essential to note that these results do not undermine the importance of frailty, but rather highlight the need to consider the mediating role of treatment limitations carefully. Despite these methodological nuances, this finding holds clinical significance. It underscores the ethical complexities inherent in using frailty to guide treatment limitations. Indeed, while it can be medically and ethically justifiable to adjust care based on a patient's frailty, we should guard against the risk of frailty becoming a self-fulfilling prophecy [21]. Our findings emphasize the critical need for a balanced approach in clinical practice that recognizes the genuine concerns related to frailty, but is also cautious not to let these perceptions overly limit the care we provide. Achieving this equilibrium is essential to circumvent potential harm that could emerge from overly conservative treatment limitations, ensuring that critically ill elderly patients receive care that is best suited to their individual needs. Furthermore, these insights call for introspection within the scientific community. How we model and decode these intricate relationships in our research can profoundly influence and shape clinical practice.

While our findings shed light on the relationship between frailty and 30-day mortality, it is pivotal to recognize the inherent limitations in using this as our sole endpoint. Specifically, 30-day mortality might not capture the broader and possibly more sustained impacts of frailty on long-term mortality and functional status. The latter, in particular, is crucial for elderly patients, as frailty at baseline might significantly affect their quality of life, daily activities, and overall well-being over time. Thus, although our study provides essential insights into short-term outcomes, a more comprehensive understanding of the implications of frailty would necessitate investigations into its effects on long-term mortality and functional status. Another significant limitation is the lack of information regarding pre-admission algorithms. This data could offer a more nuanced understanding of patient selection and management prior to ICU admission, which in turn might influence outcomes post-admission.

In our study, we found that when frailty was evaluated as a binary variable (CFS > 4), it showed a clear association with 30-day mortality, with the probability of an odds ratio (OR) greater than 1 being over 99%. However, the likelihood of this association being substantial enough to exceed 1.5 times the minimal clinically important difference (MCID) was minimal. Conversely, when the Clinical Frailty Scale (CFS) was treated as a continuous variable, the probability of observing a significantly large effect, one that surpasses 1.5 times the MCID, was markedly higher. These results highlight an essential insight: frailty, particularly when assessed using the CFS, may be more accurately represented as a continuous spectrum rather than a simple binary category. This observation was further reinforced in our sensitivity analyses and after adjusting for comorbidities, where the association of CFS with outcomes exceeding the MCID was even more pronounced, thus strengthening this aspect of our findings. This is in line with another analysis conducted by our group [22]. This continuous perspective can offer a richer, more nuanced understanding of the risks and associations, aiding clinicians in making more refined judgments and decisions. However, under all evaluated conditions, frailty fell outside the ROPE, which supports the clinical relevance of frailty in either definition—as a continuous or a binary variable.

## Conclusion

This study reinforces the significance of frailty as an independent predictor of short-term mortality in critically ill elderly patients. The distinction between viewing frailty as a dichotomy versus a continuum is apparent. While the effect size was significant when frailty was viewed as a binary factor (CFS ≥ 5), the continuous interpretation of the CFS highlighted a more pronounced effect, surpassing 1.5 times the MCID. This underscores the importance of interpreting frailty as a continuum to understand its clinical implications better. Hence, the CFS, especially when used as a continuous measure, can serve as a vital instrument for enabling risk-stratified, individualized care for this susceptible group.

## Data Availability

Upon receiving specific requests, we will provide access to the data.

## References

[1] Cohen JE (2003). Human population: the next half century. Science.

[2] Jung C, Guidet B, Flaatten H (2022). Frailty in intensive care medicine must be measured, interpreted and taken into account!. Intensive Care Med.

[3] Rockwood K, Song X, MacKnight C (2005). A global clinical measure of fitness and frailty in elderly people. CMAJ.

[4] Vallet H, Guidet B, Boumendil A (2023). The impact of age-related syndromes on ICU process and outcomes in very old patients. Ann Intensive Care.

[5] Guidet B, Flaatten H, Boumendil A (2018). Withholding or withdrawing of life-sustaining therapy in older adults (≥ 80 years) admitted to the intensive care unit. Intensive Care Med.

[6] Jung C, Flaatten H, Fjølner J (2021). The impact of frailty on survival in elderly intensive care patients with COVID-19: the COVIP study. Crit Care.

[7] Bruno RR, Wernly B, Bagshaw SM (2023). The clinical frailty scale for mortality prediction of old acutely admitted intensive care patients: a meta-analysis of individual patient-level data. Ann Intensive Care.

[8] Flaatten H, Guidet B, Andersen FH (2021). Reliability of the clinical frailty scale in very elderly ICU patients: a prospective European study. Ann Intensive Care.

[9] Beil M, van Heerden PV, de Lange DW (2023). Contribution of information about acute and geriatric characteristics to decisions about life-sustaining treatment for old patients in intensive care. BMC Med Inform Decis Mak.

[10] Pasricha V, Gorman D, Laothamatas K (2020). Use of the serious illness conversation guide to improve communication with surrogates of critically Ill patients. A Pilot Study ATS Sch.

[11] Flaatten H, De Lange DW, Morandi A (2017). The impact of frailty on ICU and 30-day mortality and the level of care in very elderly patients (≥ 80 years). Intensive Care Med.

[12] Guidet B, de Lange DW, Boumendil A (2020). The contribution of frailty, cognition, activity of daily life and comorbidities on outcome in acutely admitted patients over 80 years in European ICUs: the VIP2 study. Intensive Care Med.

[13] Bland JM, Altman DG (2000). Statistics notes. The odds ratio. BMJ.

[14] Zampieri FG, Casey JD, Shankar-Hari M (2021). Using bayesian methods to augment the interpretation of critical care trials. An overview of theory and example reanalysis of the alveolar recruitment for acute respiratory distress syndrome trial. Am J Respir Crit Care Med.

[15] Zampieri FG, da Costa BR, Vaara ST (2022). A Bayesian reanalysis of the standard versus accelerated initiation of renal-replacement therapy in acute kidney injury (STARRT-AKI) trial. Crit Care.

[16] Makowski D, Ben-Shachar MS, Chen SHA (2019). Indices of effect existence and significance in the bayesian framework. Front Psychol.

[17] Kruschke JK, Liddell TM (2018). Bayesian data analysis for newcomers. Psychon Bull Rev.

[18] Cohen J (2013). Statistical Power Analysis for the Behavioral Sciences.

[19] van Diepen M, Ramspek CL, Jager KJ (2017). Prediction versus aetiology: common pitfalls and how to avoid them. Nephrol Dial Transplant.

[20] Guidet B, de Lange DW, Flaatten H (2018). Should this elderly patient be admitted to the ICU?. Intensive Care Med.

[21] Holm S, Warrington DJ (2023). Frailty as a priority-setting criterion for potentially lifesaving treatment-self-fulfilling prophecy, circularity, and indirect discrimination?. Camb Q Healthc Ethics.

[22] Fronczek J, Polok K, de Lange DW (2021). Relationship between the clinical frailty scale and short-term mortality in patients ≥ 80 years old acutely admitted to the ICU: a prospective cohort study. Crit Care.

